# A 90–100 GHz SiGe BiCMOS 6-Bit Digital Phase Shifter with a Coupler-Based 180° Unit for Phased Arrays

**DOI:** 10.3390/mi16091056

**Published:** 2025-09-16

**Authors:** Hongchang Shen, Hongyun Zhang, Yuqian Pu, Chong Wang, Bing Li, Xusheng Tang, Xinxi Zeng, Jiang Luo

**Affiliations:** 1 School of Cyber Science and Engineering, Southeast University, Nanjing 210096, China; fxq403702@163.com (H.S.);; 2 Nanjing Guobo Electronics Co., Ltd., Nanjing 211153, China; 3 School of Electronics and Information Engineering, Hangzhou Dianzi University, Hangzhou 310018, China; 4 School of Integrated Circuits, Southeast University, Nanjing 210096, China; 5 School of Mechanical Engineering, University of Science and Technology, Beijing 100083, China; zengxinxi@ustb.edu.cn

**Keywords:** digital phase shifter, switch-type phase shifter, broadband coupler, W-band, SiGe BiCMOS

## Abstract

This paper presents a 90–100 GHz wideband digital phase shifter with a fine resolution of 5.625°, implemented in a 0.13 μm SiGe BiCMOS process. A switch-type architecture with six cascaded units, including a novel 180° cell based on a broadband coupler, enables full 0–360° phase coverage while improving phase accuracy, bandwidth, and process robustness. Post-layout simulations demonstrate an insertion loss below 15.5 dB, an RMS phase error under 2.3°, and an RMS amplitude error better than 0.9 dB across the 90–100 GHz band. The total chip area, including test pads, is 0.39 mm^2^, making the design compact and well suited for high-density phased-array applications.

## 1. Introduction

W-band (75−110 GHz) holds significant potential for applications in wireless communications, radar detection, and biomedical imaging due to its abundant spectral resources, large available bandwidth, and low atmospheric attenuation [1,2,3]. However, at W-band frequencies, millimeter-wave (mm-wave) circuits face several challenges, including pronounced parasitic effects, high losses, degraded noise figure, limited output power, and more. Phased array antenna technology provides an effective solution to these issues by enhancing signal-to-noise ratio through signal combining and enabling agile beamforming and scanning. Phase shifters play a critical role in phased array systems by adjusting the phase of each antenna element, thereby enabling precise beam steering in various directions. With the advancement of silicon-based processes, SiGe BiCMOS technology has emerged as a promising solution for mm-wave systems, offering CMOS logic compatibility and cost-effective mass production.

Common types of phase shifters include reflective phase shifters (RPS) [4,5,6,7], vector-sum phase shifters (VMPS) [8,9,10], and switch-type phase shifters (STPS) [11,12,13]. A RPS typically consists of a quadrature coupler and two tunable loads. The phase difference between the input and isolated ports is controlled by tuning the impedances of the through and coupled ports. However, impedance matching constraints often limit the tuning range. VMPS, which comprise I/Q signal generators, amplitude control, and logic modules, achieve phase shifts by adjusting the I/Q amplitude ratio. However, they suffer from limited linearity, temperature-sensitive phase errors, and unidirectional operation, restricting their applicability [10,14]. STPS rely on *LC* filter networks and switching transistors to toggle between reference and phase-shifted states [11,15]. Compared to RPS and VMPS, STPS offer high linearity, wide bandwidth, precise phase shifts, and zero static power consumption, making them well-suited for mm-wave phased arrays [15,16,17,18,19].

Among these, the 180° phase shifter unit is the most critical component in an STPS, as it has the largest phase shift and has a significant impact on phase accuracy, amplitude variation, and bandwidth. Several 180° STPS structures have been proposed, including low-pass π-type, differential inversion-type, and high/low-pass type structures. The low-pass π-type, which cascades two 90° units, is simple and wideband but incurs larger chip area and amplitude/phase errors [14,17]. The differential inversion-type structure, suitable only for differential architectures, inverts phase by controlling switching transistors, but its performance is sensitive to input impedance and parasitic effects [11]. High/low-pass structures use phase-leading and lagging networks, switched to produce a 180° phase difference. While offering high accuracy and simplicity, they suffer from limited bandwidth and reduced robustness due to parasitic effects and process variations at higher frequencies [20,21,22,23,24].

In this work, we present a W-band 6-bit switched-type phase shifter implemented in a 0.13 μm SiGe BiCMOS process. The proposed 180° unit adopts a coupler-based low-pass architecture, replacing the conventional *LC* high-pass network with a broadband coupler. This approach improves phase accuracy, extends bandwidth, and enhances robustness against process variations. Simulation results demonstrate that the proposed phase shifter achieves a 360° phase shift range with 5.625° resolution, an RMS phase error below 2.3°, and an RMS amplitude error below 0.9 dB across 90−100 GHz. The rest of this article is organized as follows. Section 2 describes the analysis and design of the proposed shifter unit with the proposed broadband coupler. Section 3 presents and analyzes the circuit design. The simulated results that verify the design method are discussed in Section 4. Finally, the paper is concluded in Section 5.

## 2. 180° Phase Shifter with a Broadband Coupler

### 2.1. A. Design of Broadband Coupler

**Figure 1:**
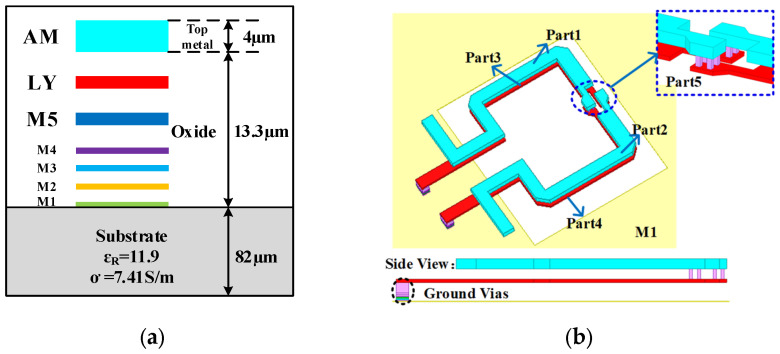
(**a**) Schematic cross-section of 0.13 μm SiGe BiCMOS process; (**b**) physical 3D structural modeling of the proposed coupler.

A broadband coupler is employed to implement a broadband 180° phase-shifted unit instead of the high-pass network in the conventional high-low-pass type structure. The proposed coupler was designed using a 0.13 μm SiGe BiCMOS process. A schematic cross-section of this process is shown in Figure 1a, which includes five thin metal layers (M1 to M5) and two thick metal layers (LY and AM). In addition, the substrate’s height, dielectric constant, and conductivity are 82 µm, 11.9, and 7.41 S/m, respectively.

The three-dimensional physical structure model of the broadband coupler is shown in Figure 1b. It mainly consists of two vertical metal vias, which are cross connected through the top metal layer AM and the sub-top metal layer LY. On both sides, there are two pairs of coupled transmission lines composed of AM and LY. Additionally, a rectangular defective ground plane is formed using the metal layer M1. In this structure, the coupled line composed of the sub-top metal LY is connected to the defective ground plane M1 through vertical metal vias. The physical length of the coupled line is a quarter wavelength, corresponding to an operating frequency of 95 GHz. Moreover, the defective ground plane alters the electric and magnetic field distributions around the coupled line. By adjusting the dimensions of the defective plane, the distributed inductance and capacitance of the coupler can be tuned, thereby offering an additional degree of freedom in coupler design [25].

**Figure 2:**
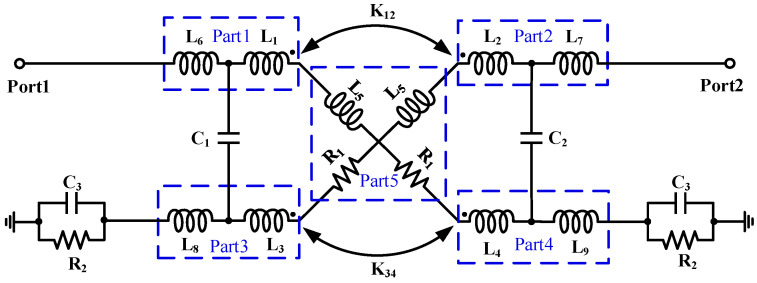
Equivalent circuit of the proposed coupler.

As shown in Figure 2, an equivalent circuit model of the proposed broadband coupler is developed using passive lumped elements, including resistance (R), inductance (L), and capacitance (C). This model enables effective evaluation of the coupler’s electrical performance when co-simulated with other circuit modules. Parts 1–4 consist of inductors L_1_–L_4_ and L_6_–L_9_ modeling the two coupled transmission lines AM and LY, respectively. R_1_ and L_5_ characterize the parasitic inductance and resistance introduced by the vertical metal via holes in Part 5. Capacitors C_1_ and C_2_ represent the coupling capacitances introduced by the AM and LY coupled transmission lines between Part 1 and Part 3, and between Part 2 and Part 4, respectively. K_12_ and K_34_ denote the coupling coefficients between Part 1 and Part 2, and between Part 3 and Part 4, respectively. C_3_ and R_2_ model the parasitic capacitance and resistance introduced by the vertical metal through-hole between the transmission line LY and the metal layer M1. They also account for substrate losses in Parts 3 & 4.

**Figure 3:**
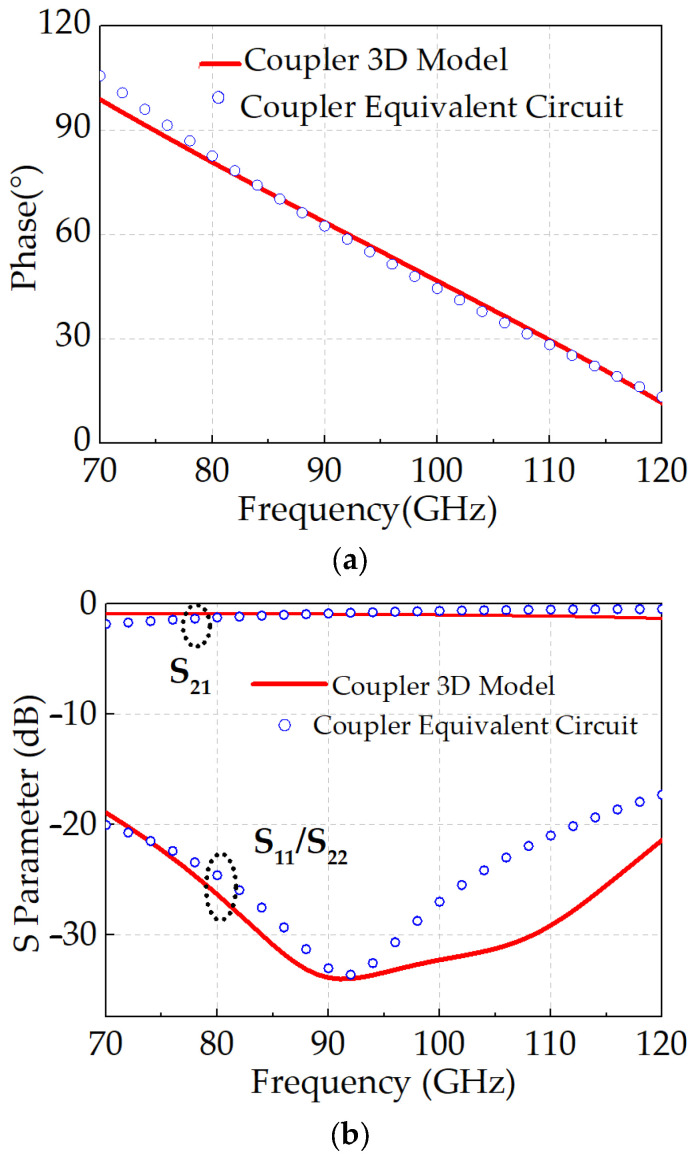
Equivalent circuit of coupler with simulation fitting curve: (**a**) phase; (**b**) S-parameters.

The simulated S-parameters and phase response of the equivalent circuit model are compared with the electromagnetic (EM) simulation results of the three-dimensional physical model, as illustrated in Figure 3. Within the frequency range of 70–120 GHz, the coupler demonstrates high-pass-like phase progression characteristics, with the transmission phase varying from 15° to 90°. The insertion loss (|S_21_|) ranges from approximately 0.8 to 1.5 dB, while the return loss (|S_11_|/|S_22_|) exceeds 19 dB.

The simulated S-parameters and phase responses derived from the equivalent circuit model exhibit excellent agreement with those obtained from full-wave EM simulations of the 3D structure. This consistency verifies the accuracy of the proposed equivalent model in predicting the coupler’s performance, facilitating further optimization of broadband coupler design. The final values of the lumped elements used in the equivalent circuit model, optimized using Advanced Design System (ADS), are summarized in Table 1.

### 2.2. B. Analysis of a Coupler-Based Low-Pass 180° Phase Shifter Unit

**Figure 4:**
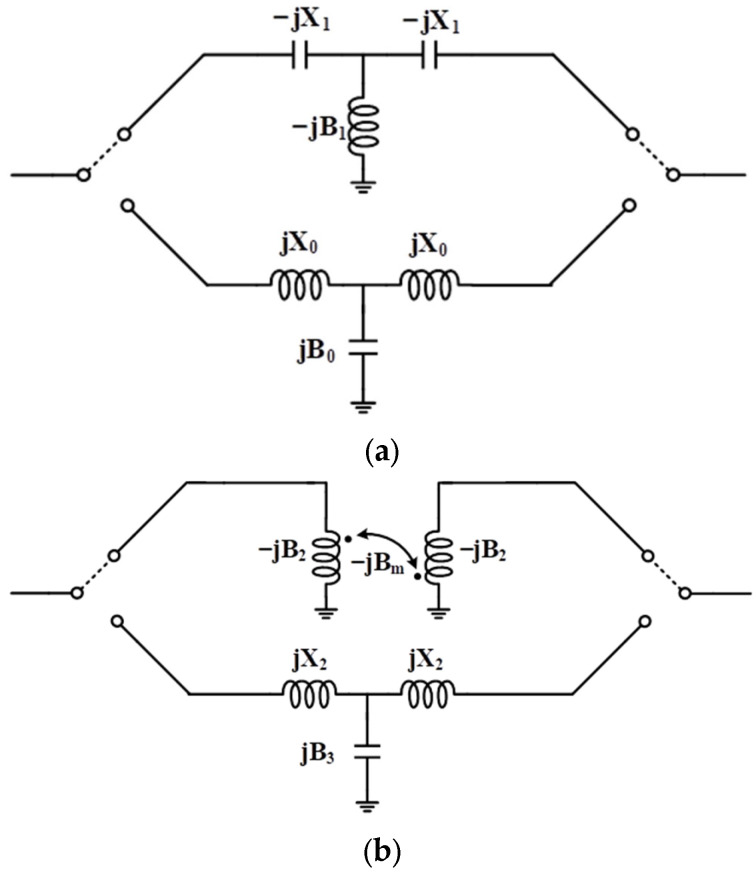
(**a**) Conventional high-/low-pass 180° unit. (**b**) Proposed coupler–low-pass 180° unit.

To improve phase accuracy and robustness in wideband applications, a coupler–low-pass configuration is adopted for the 180° phase-shifting unit. This section presents a detailed theoretical analysis comparing the proposed structure to the conventional high-/low-pass phase shifter. Figure 4a and Figure 4b illustrate the conventional high-/low-pass phase shifter structure and the proposed coupler–based low-pass configuration, respectively. The corresponding simplified equivalent circuit models for both implementations are also shown. In these models, the normalized impedances of the inductors and capacitors are denoted as X_0_, X_1_, and X_2_, while the normalized admittances are represented by B_0_, B_1_, B_2_, B_3_, and B_m_.

In the conventional high-/low-pass configuration, when the input and output ports are ideally matched to 50 Ω, the phase difference between the high-pass and low-pass networks can be expressed as

(1) $$△\phi_{1}\;=\;\tan^{-1}\left(\frac{2X_{0}}{X_{0}^{2}\;-\;1}\right)\;+\;\tan^{-1}\left(\frac{2X_{1}}{X_{1}^{2}\;-\;1}\right)$$

Here, the impedance and admittance components are defined as

(2) $$X_{0}\;=\;\tan\left(\frac{\phi_{l}}{2}\right),X_{1}\;=\;\tan\left(\frac{\phi_{h}}{2}\right),\;B_{0}\;=\;\sin\phi_{l},\;B_{1}\;=\;\sin\phi_{h}$$

Taking the derivative of (1) with respect to angular frequency ω, the phase frequency slope becomes:

(3) $$\frac{d△\phi_{1}}{d\omega }\;=\;\frac{2X_{1}}{\omega \left(X_{1}^{2}\;+\;1\right)}\;-\;\frac{2X_{0}}{\omega \left(X_{0}^{2}\;+\;1\right)}$$

For the proposed coupler–low-pass structure, the equivalent transmission matrix [ABCD] of the coupler is expressed as

(4) $$\left[\begin{array}{cc} A & B\\ C & D \end{array}\right]\;=\;\left[\begin{array}{cc} \frac{B_{2}}{B_{m}} & \frac{j\left(B_{m}^{2}\;-\;B_{2}^{2}\right)}{B_{m}}\\ \frac{j}{B_{m}} & \frac{B_{2}}{B_{m}} \end{array}\right]\;=\;\left[\begin{array}{cc} k & \frac{j\left(1\;-\;k^{2}\right)B_{2}}{k}\\ \frac{jk}{B_{2}} & k \end{array}\right]\left(B_{m}\;=\;\frac{1}{k}B_{2}\right)$$

Assuming ideal matching, the phase difference between the low-pass and coupler paths becomes:

(5) $$△\phi_{2}\;=\;\tan^{-1}\left(\frac{1}{B_{2}}\right)\;+\;\tan^{-1}\left(\frac{2X_{2}}{X_{2}^{2}\;-\;1}\right)$$

Its corresponding phase–frequency slope is

(6) $$\frac{d△\phi_{2}}{d\omega }\;=\;\frac{B_{2}}{\omega \left(B_{2}^{2}\;+\;1\right)}\;-\;\frac{2X_{2}}{\omega \left(X_{2}^{2}\;+\;1\right)}$$

When the following condition is satisfied:

(7) $$X_{0}\;=\;X_{2},\;B_{2}\;=\;\frac{1}{X_{1}}$$

The slope difference between the two-phase curves is

(8) $$\frac{d△\phi_{2}}{d\omega }\;-\;\frac{d△\phi_{1}}{d\omega }\;=\;\frac{B_{2}}{\omega \left(B_{2}^{2}\;+\;1\right)}\;-\;\frac{2X_{1}}{\omega \left(X_{1}^{2}\;+\;1\right)}\;=\;-\frac{X_{1}}{\omega \left(X_{1}^{2}\;+\;1\right)}\;<\;0$$

Equation (8) reveals that, under identical frequency and component conditions, the coupler–low-pass structure exhibits a lower phase–frequency slope than the conventional counterpart, resulting in a flatter phase response.

**Figure 5:**
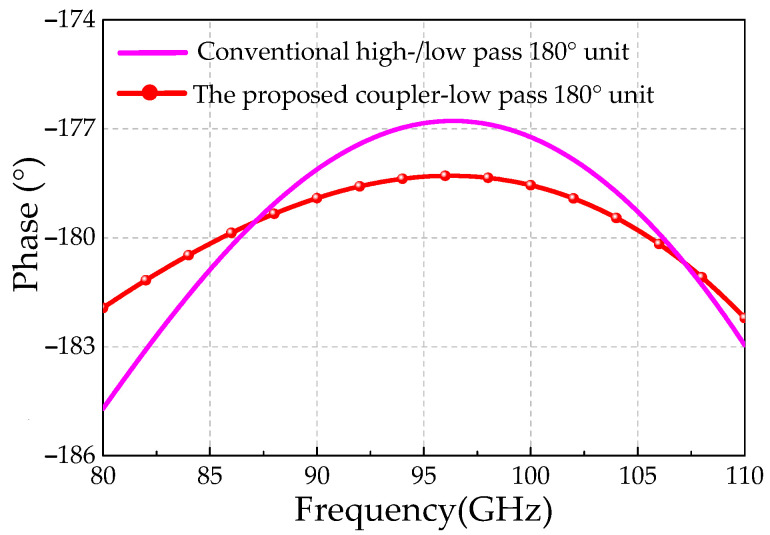
Simulated phase response comparison between the proposed coupler–low-pass phase shifter and the conventional high-/low-pass structure.

To balance insertion loss and amplitude variation, the optimized target phase shifts are set to 120° for the lagging low-pass path and approximately 60° for the leading high-pass path (including both the third-order high-pass filter and the coupler). As shown in Figure 5, the proposed 180° phase shifter achieves a variation of only 3.6° (178.3–181.9°) across 80–110 GHz, compared to 8.1° (176.6–184.7°) for the conventional structure, thus demonstrating significantly improved phase accuracy and bandwidth performance.

**Figure 6:**
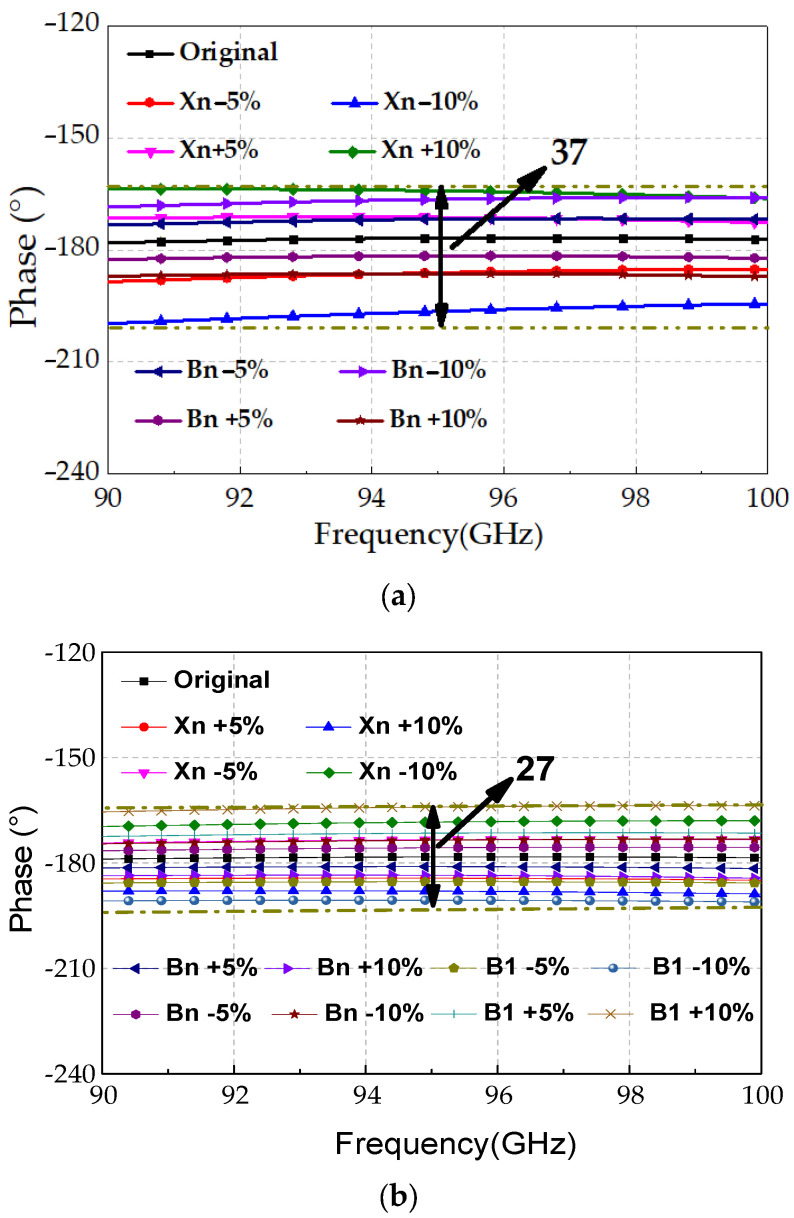
Simulated phase responses of the 180° phase-shift units under ±5% and ±10% process variations: (**a**) conventional high–low-pass-type structure; (**b**) coupler–low-pass-type structure.

Moreover, process variations have an increasing impact at millimeter-wave frequencies due to smaller passive component dimensions. Figure 6 compares phase responses under ±5% and ±10% variation in component values. Simulation results show that the proposed design limits the phase error to 27°, whereas the conventional structure exhibits a maximum deviation of 37°, yielding a 27% improvement in robustness.

## 3. W-Band 6-Bit Digital Phase Shifter Implementation

**Figure 7:**
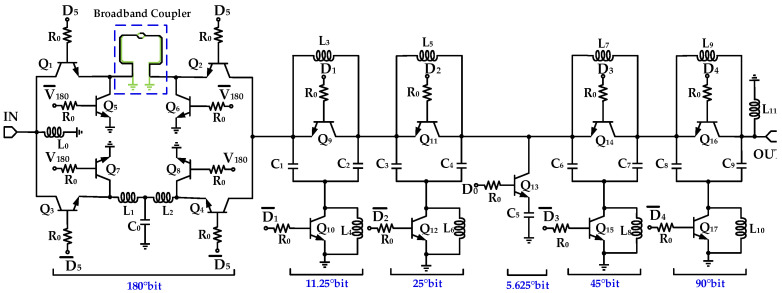
Schematic diagram of W-band 6-bit phase shifter.

The proposed W-band 6-bit digitally controlled phase shifter is composed of six cascaded unit cells providing discrete phase shifts of 5.625°, 11.25°, 22.5°, 45°, 90°, and 180°, respectively, as shown in Figure 7. Specifically, the 180° phase-shifting unit employs the proposed coupler–low-pass structure, the 5.625° unit uses a capacitor-loaded topology, and the remaining units adopt low-pass π-type configurations.

In general, each phase-shifting cell exhibits different input and output impedance characteristics in the reference and shifted states, and larger phase-shift units tend to introduce more severe impedance mismatches. To reduce impedance mismatch during phase-state switching, the 180° and 90° units are placed at both ends of the cascaded chain, while the smaller phase-shift units are arranged such that adjacent cells exhibit similar impedance transition profiles. Based on this design principle, the optimized cascade sequence is 180°-11.25°-22.5°-5.625°-45°-90°.

By independently toggling the six digital control signals D_0_, D_1_, D_2_, D_3_, D_4_, and D_5_ (corresponding to the 5.625°, 11.25°, 22.5°, 45°, 90°, and 180° bits, respectively) between 0 V and 1.2 V, the phase shifter achieves a 0–360° continuous tuning range with a least significant phase step of 5.625°. A total of 2^6^ = 64 discrete phase states are realized with this configuration.

**Figure 8:**
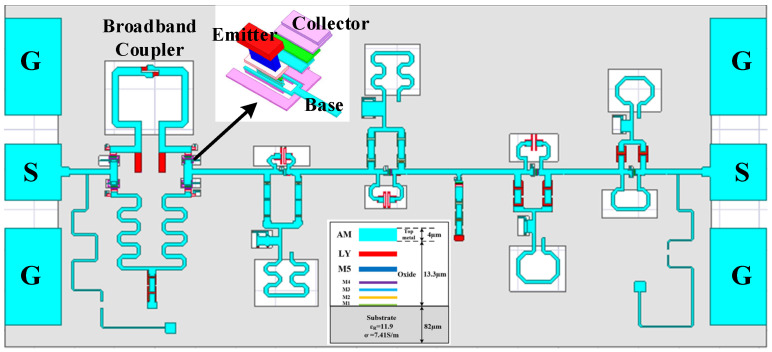
The 3-D physical model of the proposed W-band phase shifter.

In order to achieve more accurate performance of the proposed phase shifter, a 3-D physical model that incorporates all passive components—including couplers, inductors, capacitors, transmission lines, vias, interconnects, and other passive structures—was constructed in a full-wave electromagnetic (EM) simulator high-frequency structure simulator (HFSS), as shown in Figure 8. The passive structures were carefully optimized using the 3-D EM simulations, and then co-simulated together with the active devices to ensure precise circuit-level performance. The final design parameters of key devices, including transistor dimensions and critical passive components, are summarized in Table 2.

**Figure 9:**
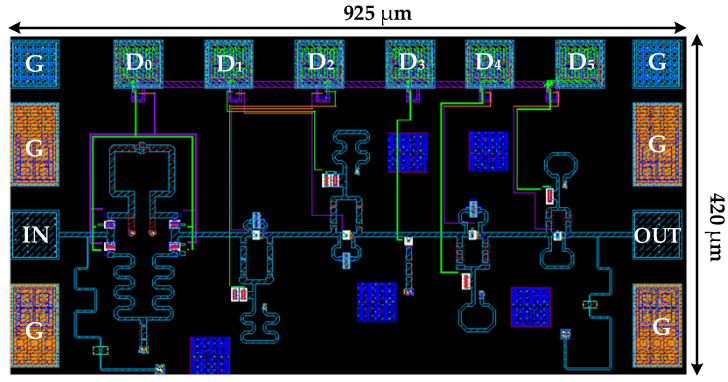
The layout of the proposed W-band phase shifter.

The chip implementation of the phase shifter was carried out in a 0.13 μm SiGe BiCMOS process. Figure 9 shows the layout of the W-band 6-bit phase shifter. Bond pads D_0_–D_5_ correspond to the control pads for the 5.625°, 11.25°, 22.5°, 45°, 90°, and 180° phase-shift units, each pad having dimensions of 100 µm × 100 µm. Ground–signal–ground (GSG) pads are used at both the input and output ends for the millimeter-wave signals. The overall die size, including all test pads, is 925 µm × 420 µm.

## 4. Results and Discussion

Based on this co-simulation framework, the post-layout simulation results and performance evaluation of the proposed W-band 6-bit digitally controlled phase shifter are presented below.

**Figure 10:**
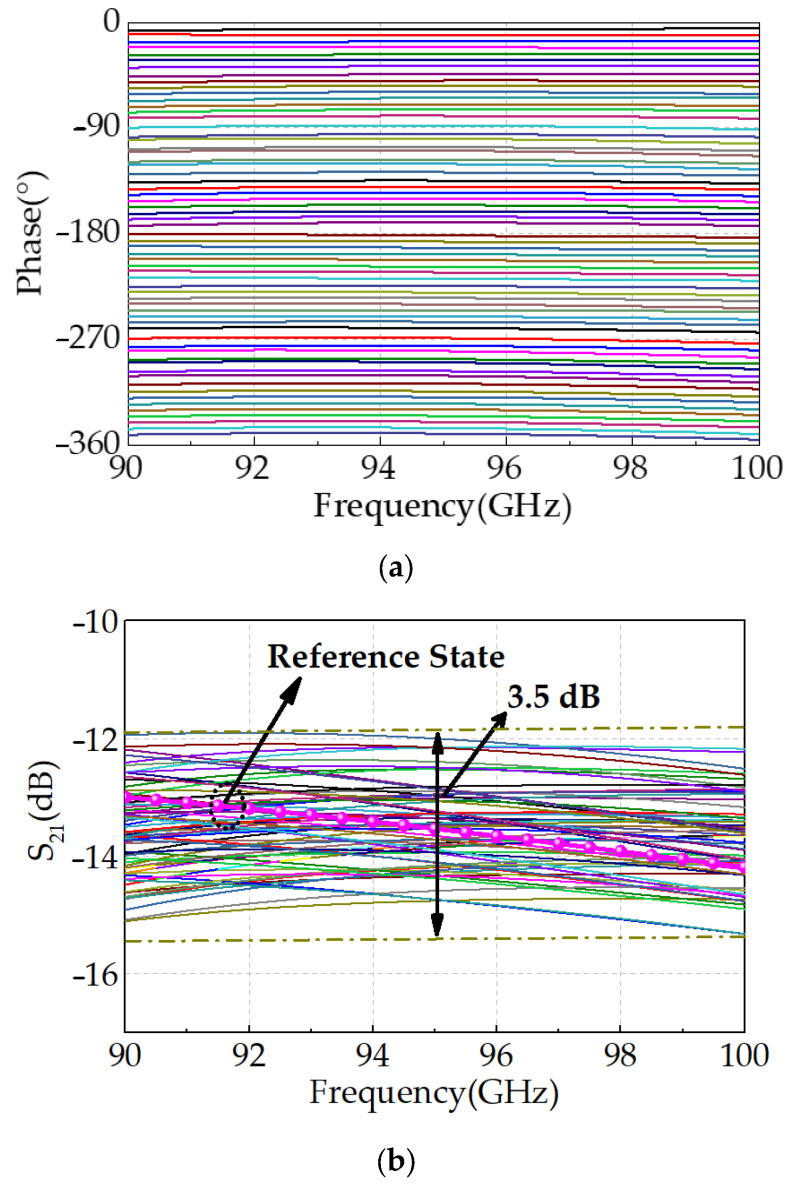
(**a**) Phase–frequency response curves and (**b**) amplitude–frequency response curves for all 64 states.

**Figure 11:**
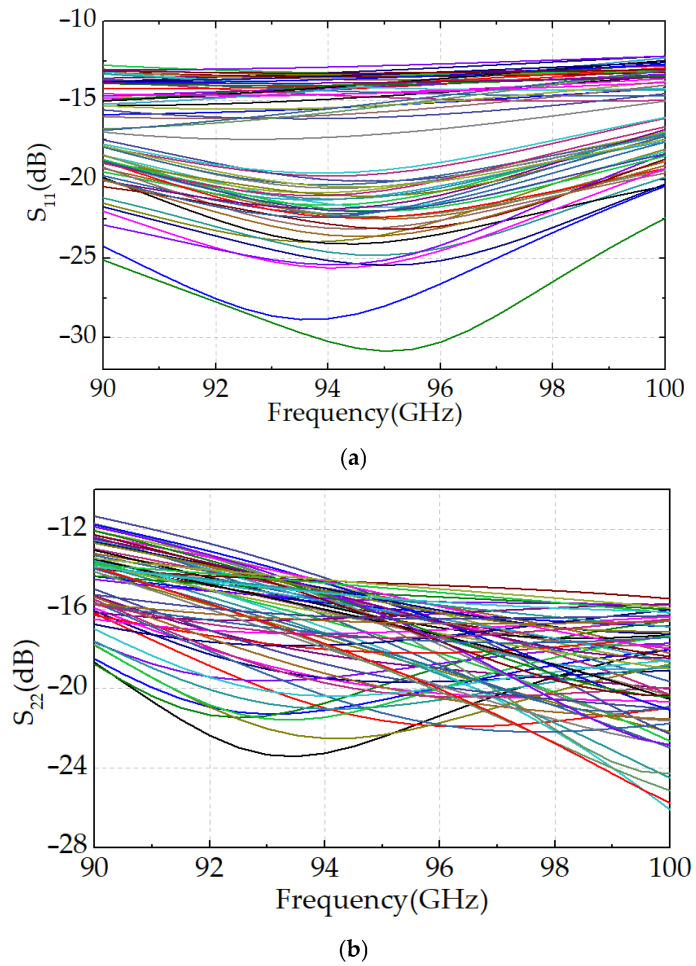
(**a**) Input return loss curves and (**b**) output return loss curves for all 64 states.

The phase–frequency response curves for all 64 states of the proposed 6-bit digital phase shifter are presented in Figure 10a. Across the target frequency range of 90–100 GHz, the curves exhibit no overlap, and the phase steps are uniformly distributed from 0° to 360° with a resolution of 5.625°, demonstrating precise and monotonic phase control. As illustrated in Figure 10b, the insertion loss (|S_21_|) ranges from 12 dB to 15.5 dB across all phase states, with a peak-to-peak amplitude variation of only ±1.75 dB relative to the reference state. The phase shifter also exhibits excellent broadband impedance matching, with both input return loss (|S_11_|) and output return loss (|S_22_|) exceeding 11 dB for all states, as shown in Figure 11.

**Figure 12:**
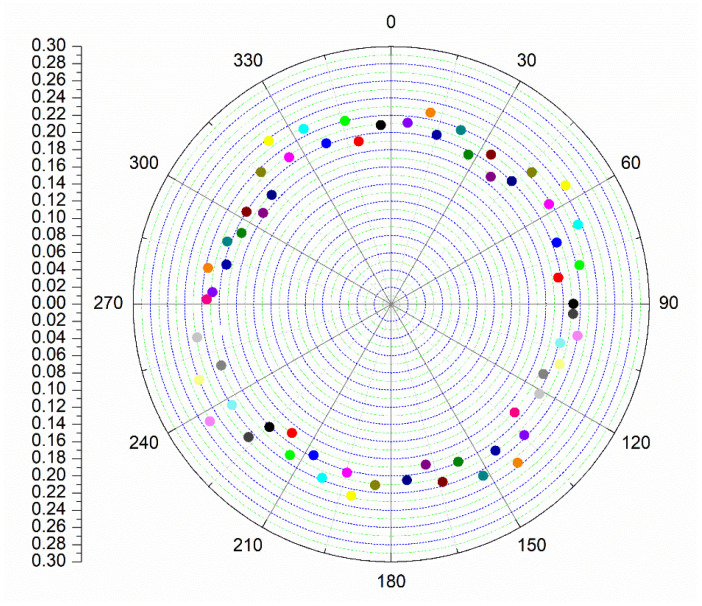
Phase and amplitude characteristics of the proposed phase shifter at 95 GHz.

To provide a clearer view of the tuning capability, Figure 12 summarizes the phase steps across all states at the center frequency of 95 GHz. Each dot corresponds to the relative phase shift and amplitude variation with respect to the reference state. The results show that the proposed design achieves a complete 360° phase coverage, while the amplitude variation across all phase states is tightly constrained within 0.23 dB, demonstrating excellent phase resolution and amplitude consistency.

**Figure 13:**
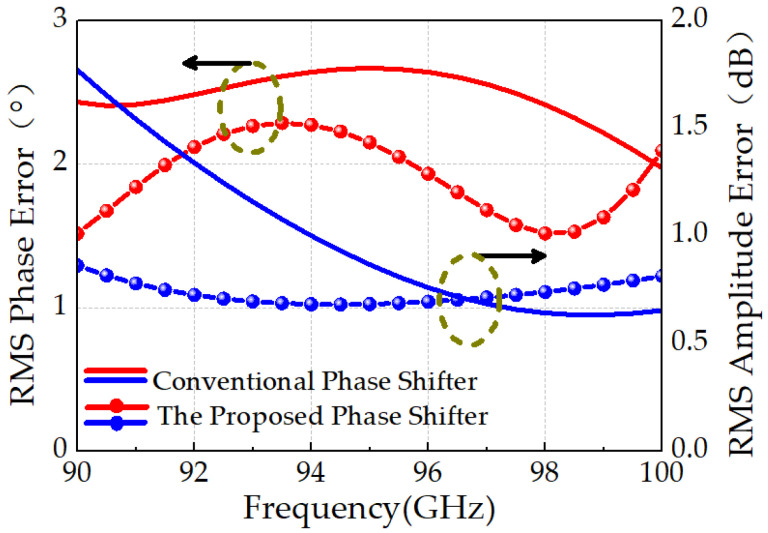
Comparison of RMS amplitude and phase errors between the coupler–low-pass-type phase shifter and the conventional high–low-pass-type phase shifter.

Thanks to the proposed coupler–low-pass structure employed in the 180° phase-shifting unit, the phase shifter achieves excellent linearity and amplitude consistency. As shown in Figure 13, the root-mean-square (RMS) phase error ranges from 1.5° to 2.3°, and the RMS amplitude error varies from 0.6 dB to 0.9 dB over the 90–100 GHz band. In contrast, conventional high-/low-pass-based 180° phase shifters exhibit significantly worse performance, with RMS phase error ranging from 1.9° to 2.7° and RMS amplitude error from 0.6 dB to 1.8 dB. Overall, the proposed W-band phase shifter achieves a 15% reduction in RMS phase error and a 50% reduction in RMS amplitude error compared to the conventional design, validating the efficacy of the coupler-assisted topology for broadband high-resolution phase control.

**Figure 14:**
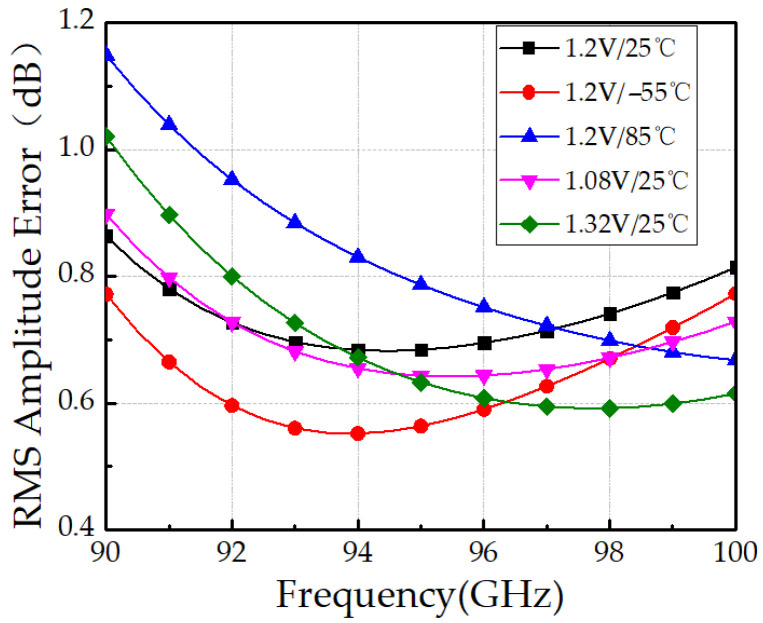
Simulated RMS amplitude errors under voltage and temperature variations.

**Figure 15:**
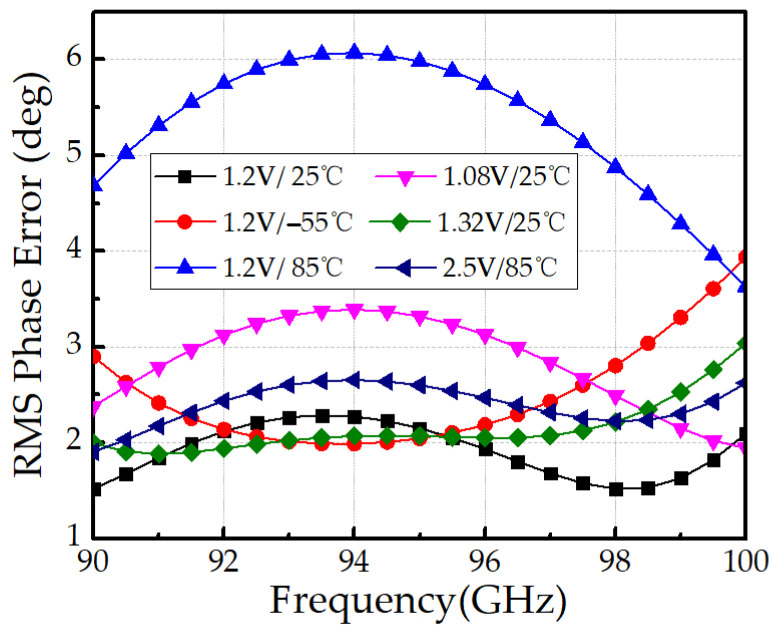
Simulated RMS phase errors under voltage and temperature variations.

We further performed post-layout simulations under operating voltage and environment temperature variations to validate the robustness of the proposed design. Specifically, (1) ±10% control voltage variation and (2) a wide temperature ranges from –55 °C to 85 °C were considered. As shown in Figure 14, the RMS amplitude error remains below 1.2 dB under these conditions, indicating strong robustness against voltage and temperature variations. However, as depicted in Figure 15, the RMS phase error degrades at high temperature, reaching 6.1° at 85 °C, mainly due to the increased ON-resistance of the HBT switch, which is inherently temperature-dependent.

To mitigate this limitation, an adaptive control voltage scheme is proposed to stabilize the ON-resistance against temperature variation. This method has been validated in our prior work [26]. As shown in Figure 14, applying a 2.5 V adaptive control voltage significantly improves the high-temperature performance, reducing the RMS phase error at 85 °C to better than 2.65°.

The performance metrics of the proposed W-band phase shifter chip are summarized in Table 3 and compared with those of recently reported silicon-based millimeter-wave phase shifters. The results demonstrate that the proposed design delivers excellent overall performance across multiple dimensions, achieving low insertion loss, high phase accuracy, and low RMS amplitude error. In particular, the incorporation of the coupler-based 180° phase-shifting unit significantly enhances both phase accuracy and process tolerance. Although Reference [6] reports the lowest RMS amplitude error among the listed works, its operating frequency lies in the V-band (50–75 GHz), which imposes fewer design challenges than the W-band. In contrast, the proposed design maintains highly competitive performance under more demanding high-frequency conditions, validating its suitability for next-generation high-resolution phased array applications.

## 5. Conclusions

A 90–100 GHz 6-bit digital phase shifter has been presented in this paper, implemented using a 0.13 μm SiGe BiCMOS process. A novel coupler–low-pass-based 180° phase-shifting unit is introduced to replace the conventional high-/low-pass topology, significantly reducing phase and amplitude errors while improving robustness against process variations. Post-layout simulation results demonstrate that the proposed phase shifter achieves a full 0–360° phase tuning range with a fine resolution of 5.625°, insertion loss below 15.5 dB, RMS phase error under 2.3°, and RMS amplitude error better than 0.9 dB across the entire 90–100 GHz frequency band. The total chip area, including all test pads, is only 0.39 mm^2^, highlighting its suitability for compact, high-density phased array systems in advanced W-band applications.

To further validate the proposed architecture, chip fabrication and on-wafer measurements are planned as part of our future work. These results will provide experimental verification of the design and confirm its applicability in practical W-band phased-array systems.

## Figures and Tables

**Table 1:** Parameter values of the components of the equivalent circuit.

L_1_	L_2_	L_3_	L_4_	L_5_
120 pH	115 pH	92 pH	92 pH	15 pH
L_6_	L_7_	L_8_	L_9_	R_1_
38 pH	39 pH	44 pH	43 pH	2.4 ohm
R_2_	C_1_	C_2_	C_3_	*K*_12_, *K*_34_
2.4 ohm	24.9 *f*F	2.7 *f*F	12.8 *f*F	0.77

**Table 2:** Key device parameter values for W-band phase shifter.

Q_1,2,3,4_ (W/L)	Q_5,6,7,8_ (W/L)	Q_9_ (W/L)	Q_10_ (W/L)	Q_11_ (W/L)	Q_12_ (W/L)
120 nm/8 µm	120 nm/1 µm	120 nm/4 µm	120 nm/17 µm	120 nm/2.5 µm	120 nm/12 µm
Q_13_ (W/L)	Q_14_ (W/L)	Q_15_ (W/L)	Q_16_ (W/L)	Q_17_ (W/L)	R_0_
120 nm/3 µm	120 nm/6 µm	120 nm/18 µm	120 nm/4 µm	120 nm/6 µm	5.7 k ohm
L_0_	L_1_	L_2_	L_3_	L_4_	L_5_
75 pH	63 pH	20 pH	65 pH	36 pH	72 pH
L_6_	L_7_	L_8_	L_9_	L_10_	L_11_
45 pH	77 pH	70 pH	85 pH	70 pH	65 pH
C_0_	C_1,2_	C_3,4_	C_5_	C_6,7_	C_8,9_
9 *f*F	5 *f*F	6 *f*F	5 *f*F	13 *f*F	17 *f*F

Note: W = emitter width of the HBT; L = emitter length of the HBT.

**Table 3:** Performance summary and comparison with state-of-the-art phase shifters.

	[14]	[11]	[12]	[16]	[15]	^★^ This Work
**Process**	90 nm CMOS	65 nm CMOS	65 nm CMOS	130 nm BiCMOS	32 nm CMOS SOI	130 nm BiCMOS
**Frequency (GHz)**	57~66	75~85	90~100	92~100	94~96	90~100
**Insertion Loss (dB)**	16~19	22~27	16~20	23~26	17~18	12~15.5
**Phase Range (°)/Bits**	360/4	360/4	360/6	360/5	360/5	360/6
**RMS** **phase error (°)**	<5	<11.25	<11	<5	<6	<2.3
**RMS** **amplitude error (dB)**	<0.5	<1.4	N/A	<1.8	<1	<0.9
**Chip Size (mm^2^)**	0.17	0.12	0.19	0.85	0.7	0.39

^★^ post-layout simulated results.

## Data Availability

The data presented in this work are available within the article.

## References

[1] Zhao D., Yu P., Jiang S., Gao W., He P., Liu H. (2024). W-band CMOS beamforming ICs and integrated phased-array antennas with 20+ Gb/s data rates. Sci. China Inf. Sci..

[2] Huang Y.S., Ni D.X., Zhou L., Zhao Z., Zhang C.R., Wang S., Xie Y., Liu R.Q., Mao J.F. (2023). A 1T2R heterogeneously integrated phased-array FMCW radar transceiver with AMC-based antenna in package in the W-band. IEEE Trans. Microw. Theory Tech..

[3] Li H.B., Chen J.X., Hou D.B., Hong W. (2019). A W-Band 6-Bit Phase Shifter with 7 dB Gain and 1.35° RMS Phase 375 Error in 130 nm SiGe BiCMOS. IEEE Trans. Circuits Syst. II Express Briefs.

[4] Natarajan A., Valdes-Garcia A., Sadhu B., Reynolds S.K., Parker B.D. (2015). W-Band Dual-Polarization Phased-Array Transceiver Front-End in SiGe BiCMOS. IEEE Trans. Microw. Theory Tech..

[5] Yishay R.B., Elad D. E-band reflection-type phase shifter with uniform insertion loss. Proceedings of the 2018 IEEE 18th Topical Meeting on Silicon Monolithic Integrated Circuits in RF Systems (SiRF).

[6] Guan P., Jia H., Deng W., Dong S., Huang X., Wang Z., Chi B. (2023). A 33.5–37.5-GHz Four-Element Phased-Array Transceiver Front-End with Hybrid Architecture Phase Shifters and Gain Controllers. IEEE Trans. Microw. Theory Tech..

[7] Ma W., Zou P., Bai L., Chen K. (2023). A Low-Loss and Full-360° Reflection-Type Phase Shifter for WLAN Wireless Backhaul Applications. IEEE Access.

[8] Smirnova K., van der Heijden M., Leenaerts D., Ulusoy A.Ç. 90–100 GHz 6-Bit Blixer-Based Active Phase Shifter in SiGe BiC-386 MOS. Proceedings of the 2025 IEEE 24th Topical Meeting on Silicon Monolithic Integrated Circuits in RF Systems 2025.

[9] Smirnova K., van der Heijden M., Yang X., Giannakidis K., Leenaerts D., Ulusoy A.Ç. (2023). W-Band 6-Bit Active Phase Shifter Using Differential Lange Coupler in SiGe BiCMOS. IEEE Microw. Wirel. Technol. Lett..

[10] Montaseri M.H., Singh S.P., Jokinen M., Rahkonen T., Leinonen M.E., Pärssinen A. A 270–330 GHz Vector Modulator Phase Shifter in 130 nm SiGe 390 BiCMOS. Proceedings of the IEEE European Microwave Integrated Circuits Conference 2021.

[11] Lee H.-S., Min B.-W. (2015). W-Band CMOS 4-Bit Phase Shifter for High Power and Phase Compression Points. IEEE Trans. Circuits Syst. II Express Briefs.

[12] Wu Y., Yu Y., Wang R., Zhang Q., Zhao C., Liu H., Wu Y., Kang K. A 90–100 GHz Passive Phase Shifter with Transistor-Based Capacitor-loaded Technique. Proceedings of the 2022 IEEE MTT-S International Microwave Workshop Series on Advanced Materials and Processes for RF and THz Applications.

[13] Li J.X., Meng F.Y., Ma K.X. A 220 GHz 5-Bit Differential Passive Phase Shifter in 0.13-μm SiGe BiCMOS. Proceedings of the 397 IEEE International Workshop on Electromagnetics: Applications and Student Innovation Competition.

[14] Lin Y.-H., Wang H. A low phase and gain error passive phase shifter in 90 nm CMOS for 60 GHz phase array system application. Proceedings of the IEEE MTT-S International Microwave Symposium.

[15] Sayginer M., Rebeiz G.M. A 94–96 GHz phased-array receive front-end with 5-bit phase control and 5 dB noise figure in 32 nm CMOS SOI. Proceedings of the IEEE MTT-S International Microwave Symposium (IMS).

[16] Afroz S., Koh K.-J. (2018). W-Band (92–100 GHz) Phased-Array Receive Channel with Quadrature-Hybrid-Based Vector Modulator. IEEE Trans. Circuits Syst. I Regul. Pap..

[17] Sayginer M., Rebeiz G.M. (2018). A W-Band LNA/Phase Shifter with 5-dB NF and 24-mW Power Consumption in 32-nm CMOS SOI. IEEE Trans. Microw. Theory Tech..

[18] Song Z., Yu Y., Zhao C., Zhang X., Zhu J., Guo J., Liu H., Wu Y., Kang K. A 94 GHz FMCW Radar Transceiver with 17 dBm Output Power and 6.25 dB NF in 65 nm CMOS. Proceedings of the IEEE MMT-S International Microwave Symposium.

[19] Morton M.A., Comeau J.P., Cressler J.D., Mitchell M., Papapolymerou J. (2006). Sources of Phase Error and Design Considerations for Silicon-Based Monolithic High-Pass/Low-Pass Microwave Phase Shifters. IEEE Trans. Microw. Theory Tech..

[20] Byeon C.W., Park C.S. (2017). A Low-Loss Compact 60-GHz Phase Shifter in 65-nm CMOS. IEEE Microw. Wirel. Compon. Lett..

[21] Song I.S., Yoon G., Park C.S. (2015). A Highly Integrated 1-Bit Phase Shifter Based on High-Pass/Low-Pass Structure. IEEE Microw. Wirel. Compon. Lett..

[22] Lim J.T., Song J.H., Kim J.H., Baek M.S., Park C., Kim C.Y. (2024). Low Insertion Loss CMOS Phase Shifter for Wireless Power Transfer System. IEEE Microw. Wirel. Technol. Lett..

[23] Chen L., Bai Y., Xing X. Performance of high-/low-pass phase shifter in broadband. Proceedings of the IEEE International Conference on Ultra-Wideband.

[24] Şengül M., Çakmak G., Özdemir R. (2021). Phase Shifting Properties of High-Pass and Low-Pass Mixed-Element Two-Ports. IEEE Trans. Circuits Syst. II Express Brief.

[25] Luo J., He J., Apriyana A., Feng G., Huang Q. (2017). A D-band SPST switch using parallel-stripline swap with defected ground structure. IEICE Electron. Express.

[26] Luo J., Peng Y., Cheng Q. (2025). A 10 to 15 GHz Digital Step Attenuator with Robust Temperature Tolerance Across −55 °C to 125 °C. IEEE Trans. Circuits Syst. II Express Briefs.

